# “Like before, but not exactly”: the Qualy-REACT qualitative inquiry into the lived experience of long COVID

**DOI:** 10.1186/s12889-022-13035-w

**Published:** 2022-03-28

**Authors:** Margherita Schiavi, Stefania Fugazzaro, Anna Bertolini, Monica Denti, Carlotta Mainini, Monia Allisen Accogli, Ginevra Bedogni, Daniele Ghizzoni, Otmen Esseroukh, Cecilia Gualdi, Stefania Costi

**Affiliations:** 1 Department of Health Professions, Azienda USL - IRCCS di Reggio Emilia, Via Amendola n°2, 42122 Reggio Emilia, Italy; 2 Physical Medicine and Rehabilitation Unit, Azienda Unità Sanitaria Locale-IRCCS di Reggio Emilia, Viale Risorgimento n°80, 42123 Reggio Emilia, Italy; 3 Bachelor’s degree in OT, Department of Medical and Surgical Sciences, University of Modena Reggio Emilia, Via del Pozzo n°71, 41124 Modena, Italy; 4 Physiotherapy Service, Casa di Cura Villa Verde, Viale Lelio Basso n°1, 42124 Reggio Emilia, Italy; 5 Bachelor’s degree in PT, Department of Surgery, Medicine, Dentistry and Morphological Sciences, University of Modena and Reggio Emilia, Via del Pozzo n°71, 41124 Modena, Italy; 6 Scientific Directorate, Azienda Unità Sanitaria Locale-IRCCS di Reggio Emilia, Viale Umberto I n°50, 42123 Reggio Emilia, Italy

**Keywords:** Post-acute COVID-19 syndrome, Qualitative research, COVID-19, Hospitalization, SARS-CoV-2

## Abstract

**Background:**

Post-acute sequelae of SARS-CoV-2 infection (PASC) affect millions of individuals worldwide. Rehabilitation interventions could support individuals during the recovery phase of COVID-19, but a comprehensive understanding of this new disease and its associated needs is crucial. This qualitative study investigated the experience of individuals who had been hospitalized for COVID-19, focusing on those needs and difficulties they perceived as most urgent.

**Methods:**

This naturalistic qualitative study was part of a single-center mix-method cross-sectional study (REACT) conducted in Italy during the first peak of the SARS-CoV-2 pandemic. The qualitative data collection took place through a telephone interview conducted 3 months after hospital discharge. The experience of individuals discharged after hospitalization for COVID-19 was investigated through the main research question – “Tell me, how has it been going since you were discharged?”. Two secondary questions investigated symptoms, activities, and participation. Data were recorded and transcribed verbatim within 48 h. An empirical phenomenological approach was used by the researchers, who independently analyzed the data and, through consensus, developed an interpretative model to answer the research question. Translation occurred after data was analyzed.

**Results:**

During the first peak of the COVID-19 pandemic, 784 individuals with COVID-19 were discharged from the hospitals of the Local Health Authority of the Province of Reggio Emilia (Italy); 446 were excluded due to the presence of acute or chronic conditions causing disability other than COVID-19 (n. 339), inability to participate in the study procedures (n. 56), insufficient medical documentation to allow for screening (n. 21), discharge to residential facilities (n. 25), and pregnancy (n. 5). Overall, 150 individuals consented to participate in the REACT study, and 56 individuals (60.7% male, average age 62.8 years ±11.8) were interviewed in June–July 2020, up to data saturation.

Persistent symptoms, feelings of isolation, fear and stigma, emotional distress, a fatalistic attitude, and return to (adapted) life course were the key themes that characterized the participants’ experience after hospital discharge.

**Conclusions:**

The experience as narrated by the participants in this study confirms the persistence of symptoms described in PASC and highlights the sense of isolation and psychological distress. These phenomena may trigger a vicious circle, but the participants also reported adaptation processes that allowed them to gradually return to their life course. Whether all individuals are able to rapidly activate these mechanisms and whether rehabilitation can help to break this vicious circle by improving residual symptoms remain to be seen.

**Trial registration:**

ClinicalTrials.com NCT04438239.

**Supplementary Information:**

The online version contains supplementary material available at 10.1186/s12889-022-13035-w.

## Background

SARS-CoV-2 began to spread rapidly throughout the world at the beginning of 2020. In little over a year since the day the pandemic was declared, 192 countries had been affected, with a cumulative 167,229,205 confirmed cases of COVID-19 [1]. While 80% of infected individuals have little or no symptoms, 15% manifest upper respiratory tract illness characterized by fever, cough, fatigue, and shortness of breath. Moreover, 5% of individuals affected by COVID-19 develop viral pneumonia with respiratory failure, requiring hospitalization [2, 3]. To date, millions of people have been declared recovered from the disease [1], but while most patients recover quickly, emerging evidence has highlighted the existence of post-acute sequelae of SARS-CoV-2 infection (PASC) [4]. In fact, several weeks after clinical recovery, symptoms of fatigue, joint pain, brain fog, and mood disturbances, just to name a few, have frequently been reported [5–11]. Moreover, restrictions on social participation have been observed in the follow-up of individuals [Fugazzaro, report submitted to *BMJ Open* on June 3, 2021].

To date, PASC probably affects millions of individuals worldwide. Research in this area is considered a priority so as to implement appropriate rehabilitation pathways, and the adoption of the International Classification of Functioning, Disability and Health framework has been suggested to arrive at a comprehensive understanding of this new disease and its associated needs [5]. However, this is only possible by investigating the whole experience of COVID-19 patients. Thus, a mix-method study named Rehabilitation Needs After COVID-19 Hospital Treatment (REACT) was designed to describe the persistent symptoms and impairments, limitations on activity, and restrictions in participation in social activities of those individuals who required hospitalization for COVID-19 (ClinicalTrials.gov Identifier: NCT04438239). This qualitative inquiry is part of the REACT study; it investigated the lived experience of individuals recovering from COVID-19, focusing on the needs and difficulties they perceived as most urgent.

## Methods

### Methodological framework

The REACT study is a single-center mix-method cross-sectional study whose aim was to collect data regarding the unmet rehabilitation needs lasting long beyond the clinical recovery of individuals who had been hospitalized for COVID-19 [Fugazzaro, submitted to *BMJ Open* on June 3, 2021]. It collected quantitative data regarding symptoms and impairments and limitations in ability and in participation through appropriate self-reported measures, i.e., the Medical Research Council (MRC) for dyspnea assessment [12], the Fatigue Severity Scale (FSS) [13], the Hospital Anxiety and Depression Scale (HADS) for mood disturbances [14], and the Barthel Index (BI) [15], and the Reintegration to Normal Living Index (RNLI) Italian version [16], to assess limitations in ability and participation.

The purpose of this naturalistic qualitative inquiry (qualy-REACT) is to provide knowledge and understanding of the experience of individuals who have been discharged after hospitalization for COVID-19. To achieve this, a combined inquiry approach (inductive and deductive) was adopted. To bring out from the data collected during the interviews, we investigated those themes deductively in terms of our specific interest in rehabilitation, i.e., persistent symptoms and limitations in activity and participation, when they did not emerge spontaneously during the interviews.

### Sampling procedure

All adult symptomatic individuals hospitalized for COVID-19 during the first peak of the pandemic and discharged from the hospitals of the Azienda USL-IRCCS of Reggio Emilia (Italy) were screened for eligibility. Excluded were individuals hospitalized for reasons other than COVID-19, individuals unable to participate in the study procedures (e.g., dementia, psychiatric disorders, language barriers, etc.), individuals with acute or chronic clinical conditions causing disability (e.g., recent stroke, surgical intervention, heart failure, etc.), and individuals with previous complete dependence in activities of daily living (ADLs).

All eligible individuals were sent a letter of invitation by ordinary mail to participate in the REACT mix-method study as well as written information and the consent form. The letter included the principal investigator’s request that a researcher be permitted to contact that individual by telephone. Two weeks after sending the letter, a researcher called the potentially eligible individuals, gave them any further information requested, and asked for their written informed consent to participate in the study. Individuals who did not answer the phone call after three attempts and those who explicitly stated they did not intend to participate in the study were deleted from the list.

According to the standard methodological approaches in qualitative research, the sample size for this qualitative study was established in the field during the data analyses. In accordance with the principles of naturalistic inquiry, the number of participants was not decided a priori, but according to data saturation [17].

### Data collection

The researchers reviewed the medical records of each consenting participant retrospectively to gather data regarding sociodemographic and clinical characteristics.

The qualitative data collection took place during the pandemic through a telephone interview that was conducted 3 months after hospital discharge.

During the interview, the experience of individuals who had been discharged after hospitalization for COVID-19 was investigated through one main research question whose aim was to bring out themes relevant to the study purpose, plus two secondary questions that investigated symptoms, activities, and participation, if necessary. Initially, the main question was “*Tell me about your experience”,* but after the first six interviews, conducted in the first week, the researchers realized that this question lacked a clear time frame, as the first interviewees frequently reported their experience during hospitalization or even their life experience before the disease. Following the iterative approach [18], the researchers modified the main research question to *“Tell me, how has it been going since you were discharged?”*

The two secondary questions, which did not need to be modified, were, “*Can you describe which symptoms still affect your daily life?” and “Concerning your everyday life, can you describe if there are any activities that you feel you haven’t fully recovered compared to before hospitalization?”*

### Data analysis and rigor

All the researchers received preliminary training on interviewing and text encoding methods. Subsequently, the seven researchers took turns conducting the interviews in pairs (an interviewer and a listener). Each interview was recorded and transcribed verbatim within 48 h by a member of the pair that conducted it.

The transcriptions of the first interviews were read carefully by all the researchers, who independently extracted the significant quotes that were labelled as preliminary codes. Starting from these preliminary codes, the researchers coded the data, compared the lists of codes, and applied that list of codes to the remaining transcripts. At the end of the coding process, codes were grouped into categories which represented the themes that had emerged. Data collection and analysis continued until no new themes were identified. All researchers discussed the coding and themes and agreed that data saturation had been reached. The data included the researchers’ spontaneous notes, which made explicit their awareness of the influence that previous experience and assumptions may have had on their data collection, analysis, and interpretation [19, 20].

All researchers critically discussed and reflected on the data before reaching an agreement on the final coherence of themes [21, 22]. Translation occurred after data was analyzed.

### Ethical considerations

The REACT study was approved by the local Ethics Committee (prot. 2020/0133, April 29, 2020) and registered on ClinicalTrials.com (NCT04438239). The research was conducted following the ICH E6 Guideline for Good Clinical Practice (GCP) and the principles of the Declaration of Helsinki. The qualy-REACT study is reported following the Consolidated Criteria for Explicit and Comprehensive Reporting of Qualitative Research (COREQ) checklist [23]). (Additional file 1).

## Results

**Fig. 1:**
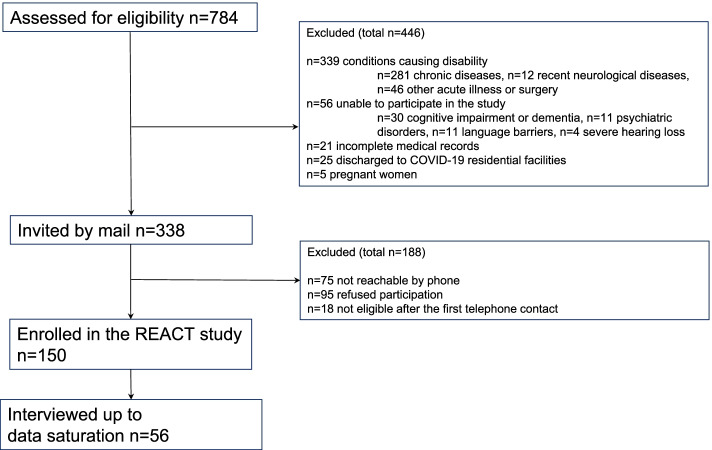
Flow diagram qualy-REACT study

From April to June 2020, 784 patients hospitalized for COVID-19 were discharged from the hospitals of the Azienda USL–IRCCS of Reggio Emilia (Italy) and screened for eligibility. Overall, 446 individuals were excluded for the following reasons: presence of acute or chronic conditions causing disability other than COVID-19 (n. 339), unable to participate in the study procedures (n. 56), insufficient medical documentation to allow for screening (n. 21). We also excluded 25 individuals who were discharged to COVID-19 residential facilities and 5 pregnant women. Thus, we sent the information by ordinary mail to 338 individuals, and called them in the following weeks. Seventy-five individuals could not be contacted, 95 refused to participate, and 18 more were excluded because they were unable to participate in the interview (sensory impairments, language barriers, etc.). Overall, 150 individuals consented to participate in the REACT study, of whom 56 were interviewed by phone (up to data saturation) in June and July 2020, at an average time of 89.9 days (±12.4) from hospital discharge. Figure 1 reports the flowchart of the qualy-REACT study.

On average, the interviews lasted a total of 30 min (including the quantitative survey). In most cases, the qualitative inquiry lasted from 15 to 20 min.

Table 1 reports the main sociodemographic and clinical characteristics of the 56 individuals who participated in the qualy-REACT study. The average age of participants was 62.8 years (±11.8); at the time of hospital admission, they were all completely independent in activities of daily living. Most participants were male (n. 34) and living with cohabitants (n. 49). Half of the sample was retired, while 26 individuals were employed. During hospitalization, 46 participants had severe COVID-19 clinical manifestations and 50 needed respiratory support. The average length of stay (LOS) was 19.1 days (±13.4) for the whole sample, and 13.7 days (±8.9) in the intensive care unit for individuals admitted to it (n. 15). At the time of the interview, very few individuals still had severe dyspnea (n.2), but several of them (n. 23) had severe fatigue. Clinically relevant anxiety and depression were reported by 14 and 9 individuals, respectively. Fifty participants were completely autonomous in basic activities of daily living, but only 13 were completely reintegrated into their normal life, and 42 individuals still had mild-to-severe participation restrictions.

**Table 1:** Characteristics of the 56 participants to the qualy-REACT study

Age, mean (SD)	62.8 (±11.8)
**Sex, N (%)**	
**Male**	34 (60.7)
**Female**	22 (39.3)
**Household composition, N (%)**	
Alone	7 (12.5)
With others	49 (87.5)
**Occupation, N (%)**	
Retired	28 (50.0)
Employed	26 (46.4)
Unemployed	2 (3.6)
**Severity of COVID-19 clinical manifestations, N (%)**	
Mild symptoms (fever, cough, fatigue, diarrhea, other)	3 (5.4)
Moderate (bilateral pneumonia)	7 (12.5)
Severe (bilateral pneumonia and respiratory failure)	46 (82.1)
Ventilatory support, N (%)	
None	6 (10.7)
Nasal cannula/face mask	28 (50.0)
Non-invasive mechanical ventilation	17 (30.4)
Intubation	5 (8.9%)
**Dyspnea (MRC)**	*(*N* = 55)
None (0)	31 (56.4)
Mild (1)	16 (29.1)
Moderate (2, 3)	6 (10.9)
Severe (4)	2 (3.6)
**Fatigue (FSS)**	*(*N* = 55)
Mild (≤ 36)	32 (58.2)
Severe (> 36)	23 (41.8)
Anxiety (HADS-a)	*(*N* = 55)
None (< 8)	41 (74.6)
Relevant (≥8)	14 (25.4)
**Depression (HADS-d)**	*(*N* = 55)
None (< 8)	46 (83.6)
Relevant (≥8)	9 (16.4)
**Independence in B-ADL (BI)**	*(*N* = 55)
Complete (100)	50 (90.9)
Mild limitation (91–99)	4 (7.3)
Moderate limitation (61–90)	1 (1.8)
Severe limitation (21–60)	0 (0.0)
**Social participation (RNLI)**	*(*N* = 55)
Complete (100)	13 (23.6)
Mild limitation (60–99)	38 (69.1)
Severe limitation (< 60)	4 (7.3)

*(*N* = 55) = objective assessments were feasible for 55 participants because one individual had difficulty understanding the questions over the phone

Legend: *SD* Standard deviation, *N* Number, *MRC* Medical Research Council, *FSS* Fatigue Severity Scale, *HADS-a* Hospital Anxiety and Depression Scale – anxiety score, *HADS-d* Hospital Anxiety and Depression Scale – depression score, *B-ADL* Basic Activities of Daily Living, *BI* Barthel Index, *RNLI* Reintegration to Normal Living Index

### Explanatory theoretical model

**Fig. 2:**
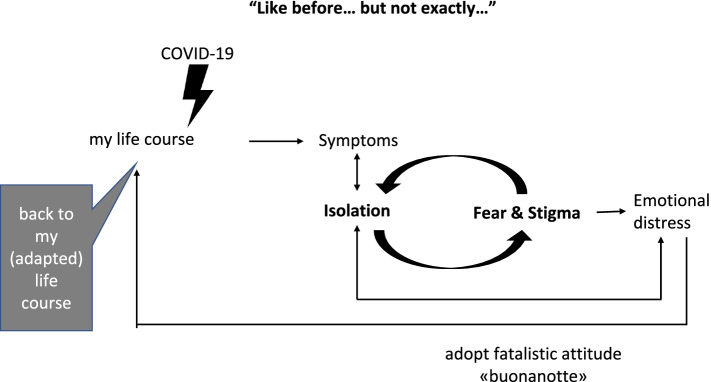
Psychosocial implications of the recovery phase from COVID-19

Six themes emerged from the data: persistent symptoms, feelings of isolation, fear and stigma, emotional distress, fatalistic attitude (“goodnight”), and return to their (adapted) life course (Fig. 2). They represent the symptoms and psychosocial implications of long COVID and are described in Table 2, together with the subthemes and exemplary quotes.

**Table 2:** Themes and subthemes that characterized the patient’s experience of long COVID

Theme and subtheme	Exemplary quotes
**Persistent symptoms**	
Respiratory symptoms	“When I take a long walk, I become short of breath” (A072, male, 63 years). “For me, aside from this breathing, which I didn’t have before …” (A029, male, 55 years) “I would go to the toilet, come back, and immediately feel I couldn’t breathe.” “I can climb the stairs now, but not without getting winded.” “I also have this cough, which is quite bothersome during the day.” (A033, male, 51 years) “I’m always breathless.” (A056, female, 47 years) “When I walk at a brisk pace, faster than necessary, I always get short of breath.” (A048, female, 31 years) “I feel the breathlessness here.” (A064, male, 61 years) “Maybe I get a little short of breath when I’m doing very physical tasks; I have to stop a minute.” (A075, female, 81 years)
Fatigue, tiredness, exhaustion	“I was really tired, exhausted, I just couldn’t do anything.” “I still get tired pretty quickly.” “I still have this sense of fatigue whenever I do anything.” (A034, male, 59 years) “I felt tired.” (A039, male, 64 years) “I don’t get winded anymore, but I’m still tired.” (A033, male, 52 years) “It’s obvious, everything is harder for me.” (A031, female, 80 years) “Anyway, there’s this constant fatigue.” (A049, female, 40 years) “Everything is harder than it used to be.” (A069, female, 49 years) “This fatigue is a bit hard.” “It takes more effort for me to do certain things” (A010, female, 58 years) “Well, exhaustion sometimes …” (A069, female, 49 years) “I’m always really tired” (A036, female, 77 years) “It also means that when I finally get to where I’m going, I don’t enjoy it because I’m so totally exhausted that I can’t” (A060, male, 73 years) “I had a bit of trouble with my legs” (A043, male, 62 years)
Strength	“I don’t have any strength.” (A031, female, 80 years) “I never recovered my strength.” (A060, male, 73 years) “My legs shook.” (A033, male, 51 years)
Muscle and joint pain	“My legs hurt so much it feels like someone is hammering them.” “My back started to hurt.” (A066, female, 56 years) “I spent a month at home with pain in my legs.” (A023, male, 66 years) “I have problems because of the pain in my arms and shoulders.” (A026, female, aged 59) “I still have a backache.” (A048, female, 32 years)
Hypogeusia and Hyposmia	“My sense of smell and taste have changed.” (A037, male, 65 years) “I can’t smell anything.” (A039, male, 64 years) “Everything tastes different, less intense. Even smells, certain smells, are different.” (A049, female, 40 years) “Sometimes my sense of taste changes a little.” (A058, male, 54 years) “The symptom I still have is this loss of smell; I smell things, but they smell different.” (A078, female, 54 years) “The only thing I still have is that I can’t taste food well; everything tastes bitter, tasteless.” (A013, female, 80 years)
Paresthesia	“I still have this problem with two fingers, which I can’t feel; I have tingling 24 h a day.” (A013, male, 49 years) “I feel even my nerves have been affected.” (A033, male,.52 years)
Hair loss	“The only thing that’s a problem now is my hair; I’m losing a lot of hair.” (A043, female, 62 years) “I lost my hair, and that was really hard for me.” (A077, male, 64 years) “And I’m losing my hair.” (A78, female, 54 years)
Hand tremors	“My hands shook.” (A012, male, 71 years)
Vision loss	“My eyesight has got worse.” (A012, male, 71 years) “My eyesight has got a bit worse.” (A021, female, 61 years)
**Feelings of isolation**	
Lockdown	“When I was discharged in early April, I couldn’t even go to the cemetery because they were all closed.” (A044, female, 62 years) “At that time, you didn’t even see anyone …” “Everyone had to deal with this hardship; you couldn’t go out, you couldn’t see your family …” (A022, female, 81 years) “In that period, what came to mind was not having seen my family for at least 20 days.” (A037, male, 65 years)
Quarantine	“If I could have hugged them, I would have hugged all of them.” (A007, male, 70 years) “I was alone in a room, I didn’t see anyone.” (A008, male, 64 years) “Maybe because of my long quarantine … I was isolated in a room for almost a month …” (A014, female, 49 years) “I had to do 14 more days of isolation.” (A013, female, 80 years) “In those moments of hardship, I thought about the fact that I hadn’t seen my family for at least 20 days.” (A037, male, 65 years)
**Fear and stigma**	
Fear of the experience	“I still feel a little afraid when I talk about it.” (A010, female, 58 years) “I always get chills, goose bumps.” (A029, male, 55 years) “I didn’t even want to watch the TV programs that talked about the number of deaths, new cases …” (A026, female, 59 years) “I can’t watch TV; it’s all they talk about.” (A060, male, 73 years)
Fear of the unnamable	“When you’re in the hospital and everyone only talked about bad things …” (A029, male, 55 years) “This thing... the more time passes, the more I realize ...” (A044, female, 62 years) “I always get chills … goose bumps … just thinking about it” (A029, male, 55 years)
Fear of infecting others	“But, as I told you, I already suspected that maybe I wasn’t well even before.” (A036, female, 77) “I’m afraid to be physically close to anyone.” (A049, female, 40 years) “Let’s just say that now I avoid going to dinners, I don’t go.” (A054, male, 53 years) “When I get out of here, I want to make sure I don’t carry the virus.” (A040, male, 78 years)
Other people’s fear of getting infected, stigma	“Get better, completely better, that way we can all rest easy.” (A029, male, 55 years) “But they ask you questions to find out if maybe they missed a symptom they had because they’re afraid.” (A037, male, 65 years) “Everyone is suspicious. Basically, everyone is afraid.” (A017, female, 71 years)
Fear of getting sick again	“I’m a bit terrified about getting sick again.” (A021, female, 61 years) “We have to be very careful.” (A022, female, 81 years) “How will I deal with the flu one day?” (A026, female, 59 years) “I’m afraid to be around people.”, “I’m afraid to catch it again … I’m afraid to go out, to see people.” (A049, female, 40 years) “Let’s just say that now I avoid going to dinners, I don’t go.” (A054, male, 53 years) “Everyone is diffident, everyone is afraid, we don’t go out; if we want a pizza, we eat it at home.” (A017, female,71 years)
**Emotional distress**	
Depression	“Then there’s this sort of depression; I don’t want to call it depression, but a lack of interest in anything, from morning to night.” (A060, male, 73 years)
Anxiety	“I even had an anxiety attack that kept me up the whole night.” (A026, female, 59 years) “I’ve been a little anxious and depressed.” (A037, male, 65 years) “My symptoms are mostly psychological, like anxiety.” (A032, male, 67 years) “This thing caused me anxiety and panic attacks, it was bad for me.” (A033, male, 52 years) “All this anxiety and paranoia” (A066, female, 56 years)
Worry	“I worry a little more than I used to.” (A058, male, 54 years)
**Fatalistic attitude**	
*“Goodnight”*	“I tell you, I always try to think positive, in the sense that I live my life. Every morning I wake up, I say to myself, “Today I woke up, which is a lot.” Some things in life just happen, you don’t go looking for them; they just happen and that’s it, *buonanotte*” (A010, male, 71 years) “If tomorrow they tell me I have to die, well, *buonanotte*” (A007, male 70 years)
**Return to (adapted) life course**	
Not like before	“I tell you, I always try to think positive, in the sense that I live my life. Every morning I wake up I say to myself, “Today I woke up, which is a lot.” Some things in life just happen, you don’t go looking for them; they just happen and that’s it, buonanotte” (A010, male, 71 years) “Making breakfast seemed like a challenge, you take out the biscuits, you make the coffee, you get out the milk, I said, “Oh for heaven’s sake!” (A026, female, 59 years)
Like before but not exactly	“Everything is back to normal. I’m calm, that is ... sometimes I have a fit of irritation for some reason, but overall, I’m fine, yeah.” (A013, female, 80 years) Now, I do everything. If I have to climb the stairs, no, but I’d like to. Yes, I do almost everything.” (A024, female, 69 years).
Adaptation	“Well, you know, well, my life, if I have to go out, do the shopping, I have a lot of friends … life goes on, unfortunately”, “You know, well, that’s how things are.” (A022, female, 81 years) “Let’s say … ok, it’s over. You keep going.” – (A023 male, 66 years) “I’d be happy just to do something … about these breathing problems.” (A033 male, 51 years) “Anyway, what I say to my friends, to the people who know me, is that I feel lucky because, overall, I’m ok.” (A036, female, 77 years) “Psychologically, I feel better than before because I didn’t use to appreciate some things, now I do, more. The sense changed a little, I want more to live, I’m happy, basically. I can’t say anything else.” (A052, male, 65 years) “Now when I talk about it, I’m a little afraid, but it all worked out well, quote-unquote well.” (A010, female, 58 years)

#### Persistent symptoms

Three months after clinical recovery from the infection, individuals still had a plethora of symptoms, several of which associated with respiratory impairment and bed rest.

“When I take a long walk, I become short of breath” (A072, male, 63 years).

Fatigue was frequently evoked, especially in females

“Everything is harder than it used to be” (A069, female, 49 years).

However, several symptoms of various nature, for example, vision loss, tremor in the hands, and hair loss, just to name a few, were also reported:

“My eyesight has got worse” (A012, male, 71 years), “My hands shook” (A012, male, 71 years), “And I’m losing my hair” (A78, female, 54 years).

### Isolation

Aside from their physical symptoms, participants also experienced isolation, which emerged in two different situations: lockdown, which limited the social activities of the general population

“When I was discharged in early April, I couldn't even go to the cemetery, because they were all closed” (A044, female, 62 years),

and the individual quarantine measures, which were necessary to avoid contact with infected individuals, which resulted in personal isolation

“...I had to do 14 more days of isolation” (A013, female, 80 years)*.*

### Fear and stigma

Fear and stigma characterized the convalescence phase. This psychosocial theme emerged as the fear of recalling and naming the experience, the fear of getting sick again, which seemed especially present in females, the fear of infecting others, and the fear of others being infected

*“*I still feel a little afraid when I talk about it” (A010, female, 58 years),

“I’m a bit terrified about getting sick again” (A021 female, 61 years),

“When I get out of here, I want to make sure I don’t carry the virus” (A040, male, 78 years). From the stories of some participants, a feeling of shame in telling others about the illness also emerged

“Everyone is suspicious. Basically, everyone is afraid” (A017, female, 71 years), as did the stigma for having had the disease

*“*Get better, completely better, that way we can all rest easy*”* (A029, male, 55 years).

Fear and stigma also manifested themselves by not naming the disease, which often remained implicit in the narratives

“This thing … the more time passes, the more I realize *…* ” (A044, female, 62 years)

“I always get chills … goose bumps … just thinking about it” (A029, male, 55 years).

### Emotional distress

Further psychosocial implications characterizing the convalescence phase from COVID-19 were feelings of anxiety, depression, and worry. Depression manifested as a loss of interest that permeated the entire day

*“*Then, there’s this sort of depression; I don’t want to call it depression, but a lack of interest in anything, from morning to night” (A060, male, 73 years), while anxiety was also accompanied by feelings of paranoia and upset, and even panic attacks

*“*This thing caused me anxiety and panic attacks, it was bad for me*”* (A033, male, 52 years).

### Fatalistic attitude

A fatalistic attitude has been summarized as the “goodnight” (*buonanotte* in Italian), an expression frequently used by the participants. It is an Italian linguistic interlayer that, in the local culture, expresses a sense of helplessness towards an uncertain future. This theme, which emerged more frequently in older males, indicated the need to resume living despite being resigned to the fact that the disease could recur at any moment,

*“*I tell you, I always try to think positive, in the sense that I live my life. Every morning I wake up, I say to myself, “Today I woke up, which is a lot.” Some things in life just happen, you don’t go looking for them; they just happen and that’s it, *buonanotte*” (A010, male, 71 years)

*“*If tomorrow they tell me I have to die, well, *buonanotte”* (A007 male, 70 years).

### Returning to (adapted) life course

Two different perceptions emerged regarding the return to normal life, and several data support a not complete recovery (not like before) with respect to work, to social and family roles, and to leisure time activities as well as, sometimes, to basic activities of daily life, such as climbing stairs or preparing breakfast

“...I do some things, but clearly not like before … I mean, do some things that before took me half a day, now it takes me four or five days*”* (A034, male, 59 years),

*"*Making breakfast seemed like a challenge, you take out the biscuits, you make the coffee, you get out the milk, I said, “Oh for heaven’s sake!” (A026, female, 59 years).

However, while other participants claimed a complete return to the activities they used to engage in before the illness, sometimes their words betrayed a hesitation with respect to this statement (like before - but not exactly)

“Everything is back to normal. I'm calm, that is ... sometimes I have a fit of irritation for some reason, but overall, I'm fine, yeah” (A013, female, 80 years)

“Now, I do everything. If I have to climb the stairs, no, but I’d like to. Yes, I do almost everything” (A024, female, 69 years).

These narratives led us to the major theme of adaptation, which was explicitly brought up by some participants, and from which their awareness of having passed through a frightening period was perceptible.

“The way I got it, I'm very happy with the way I recovered” (A019, male, 42 years)

“Now when I talk about it, I’m a little afraid, but it all worked out well, quote-unquote well” (A010, female, 58 years).

Altogether, the themes that emerged characterize the recovery phase of COVID-19 and represent the interpretative model to answer the research question “*How has it been going since you were discharged?*”

## Discussion

This study provides evidence of the experience of individuals hospitalized for COVID-19. After discharge, individuals still have a plethora of symptoms, which confirms that this disease can have long-lasting physical and psychological effects and that a careful follow-up, tailored to each individual’s health needs, is appropriate. Persistent symptoms of fatigue, dyspnea, musculoskeletal pain, and loss of muscle strength as well as hypogeusia and hyposmia have already been detected in patients with COVID-19 [10, 11, 24], but this qualitative inquiry allowed many others to emerge, such as hair loss, visual loss, paresthesia, and hand tremors. This heterogeneous scenario describes an illness trajectory which, at the time of the interviews, was not yet acknowledged by healthcare professionals, who could not give appropriate suggestions to individuals with PASC. However, even if none of these symptoms, taken individually, is likely to lead the patient to seek medical attention, taken together they can contribute to the burden of disease.

In the sample considered in this study, most patients (82%) were hospitalized for severe clinical manifestations of COVID-19 (bilateral pneumonia or respiratory failure), needing low-flow oxygen support up to high flow or mechanical ventilation. This explains the plethora of symptoms and the psychological distress reported: three months after discharge, almost half of the patients still suffered from shortness of breath and severe fatigue, and a clinically relevant level of anxiety was detected in a fourth of the sample [Fugazzaro, submitted to *BMJ Open* on June 3, 2021]. This qualitative study also allowed several symptoms of emotional distress to emerge; participants told us about experiences of depression, anxiety, worry, and particularly fear, which may be justified by the serious concerns engendered by the pandemic itself and by the restrictions imposed to contain it, as appears from a previous study [25]. However, emotional distress may also be attributed to experiencing a new disease whose course was uncertain and to the conflicting information received from healthcare professionals and the media [5]. Of note, high rates of emotional distress were also detected in SARS and MERS survivors which persisted even years post-infection [26, 27].

The data collected showed that individuals frequently talk about COVID-19 without naming it, that they are afraid to talk about their experience

*“*I always get chills … goose bumps … just thinking about it” (A029, male, 55 years)

and prefer not to watch the news on television. This ‘avoidance’ may be a sign of post-traumatic stress disorder, whose presence has been demonstrated in the post-acute phase of COVID-19 [28], particularly in individuals who had been admitted to the intensive care unit [29], as was also seen during the SARS epidemic, the first of this millennium [26]. Alternatively, avoidance may simply be the result of the fear of the disease and the associated stigma, which can be surmised from the interviewees’ words concerning their fear of getting sick again and of infecting others, again as happened during the SARS epidemic [26]. But the fear of others being infected, which leads some individuals to feeling ashamed of having had the disease and of being labelled a “disease carrier” is also reported, especially by women. This finding is in line with evidence that shows how prejudice behaviors and stigma have been concerns in previous viral outbreaks [30], which can last several months [5]. In our opinion, the stigma phenomenon is of particular importance because it can foster both social isolation and emotional distress [5]. Specific recommendations were made at the beginning of the pandemic to limit this phenomenon [31] because a vicious circle of isolation, stigma, and emotional distress can trigger and feed itself [32]. This hypothesis is corroborated by the quantitative data collected by the REACT study, which showed that despite excellent recovery of basic activities of daily living, 76% of study participants had still not recovered full social participation 3 months after hospital discharge [Fugazzaro, submitted to *BMJ Open* on June 3, 2021]. Indeed, the return to normal life must be adapted to the post-COVID-19 reality, where the feeling of not returning to one’s occupations and family role “like before” is widespread. Of note, 17% of Canadian SARS survivors had not returned to work 1 year after infection, and 44% were referred to mental health services [33]. Therefore, given that thousands of individuals are experiencing PASC worldwide, the qualitative data this study provides may be particularly timely.

### Limitations of the study

While the sample of this study made a description of the lived experience of COVID-19 survivors possible, since the interviews were conducted only once, 3 months after hospital discharge, it was not possible to observe how the recovery process evolved. Furthermore, although we followed specific techniques to ensure the trustworthiness and credibility of the data analysis, the lack of the perspective of patients who had not been hospitalized challenges the transferability of our results to other populations and/ or to different health care contexts. We cannot know whether any of the themes emerging from this study would have been confirmed by data collected from patients with mild symptoms or even from those who were asymptomatic. Future studies should investigate the personal experience of COVID-19 in a broader patient population, including individuals with mild symptoms and asymptomatic individuals, over a longer period of time. Moreover, since the data were collected during the first peak of the pandemic, the interviews were conducted over the phone. The use of telephone interviews in qualitative research is debated, as it is thought that they may lead to interpretation biases of the data collected [34]. However, there is also evidence in favor of this approach [35]. Furthermore, it made this investigation feasible without exposing the patients to risks or inconveniences. If more questions had been asked, more data would have emerged, and the lived experience of people who were discharged after hospitalization for COVID-19 would likely have been described in more depth. However, we chose to ask few questions in an effort to foster a good level of compliance in the more fragile participants, who may still have been suffering from dyspnea or fatigue.

## Conclusions

The experience as narrated by the participants in this study confirms the existence of PASC in patients who suffered severe COVID-19. The data collected allow clinicians to appreciate the subjective, not easily measurable aspects of the patients’ experience after hospital discharge. The persistence of symptoms and isolation trigger a mechanism that feeds itself through the phenomena of fear and stigma. However, individuals also activate recovery processes that allow them to gradually return to their life course. Whether all individuals are able to rapidly activate these mechanisms and whether rehabilitation can help to break this vicious circle by improving residual symptoms remain to be discovered.

## Supplementary Information

**Additional file 1.** The COREQ checklist of the report.

**Additional file 2.** Study Consent Form.

## Data Availability

Reasonable requests for all of the individual participant data collected during the trial, after deidentification, should be made to the corresponding author and will be considered by the REACT lead author. The presented data are anonymized, and risk of identification is low.

## Abbreviations

COVID: Corona virus disease
PASC: Post-acute sequelae of SARS-CoV-2 infection
REACT: Rehabilitation Needs After COVID-19 Hospital Treatment
