# Supplementary material for: “Like before, but not exactly”: the Qualy-REACT qualitative inquiry into the lived experience of long COVID
Source: BMC Public Health. 2022 Mar 28;22:599. doi: 10.1186/s12889-022-13035-w (PMC8960224; doi:10.1186/s12889-022-13035-w)
Supplement: Supplementary file 1 — Additional file 1. The COREQ checklist of the report. [file 12889_2022_13035_MOESM1_ESM.docx]

**COREQ (COnsolidated criteria for REporting Qualitative research) Checklist**

A checklist of items that should be included in reports of qualitative research. You must report the page number in your manuscript where you consider each of the items listed in this checklist. If you have not included this information, either revise your manuscript accordingly before submitting or note N/A.

| **Topic** | **Item No.** | **Guide Questions/Description** | **Reported on Page No.** |
| --- | --- | --- | --- |
| **Domain 1: Research team and reflexivity** | | | |
| *Personal characteristics* |  |  |  |
| Interviewer/facilitator | 1 | Which author/s conducted the interview or focus group? | 8 Data analysis |
| Credentials | 2 | What were the researcher’s credentials? E.g. PhD, MD | 1 Title page |
| Occupation | 3 | What was their occupation at the time of the study? | 1 Title page |
| Gender | 4 | Was the researcher male or female? | 1 Title page |
| Experience and training | 5 | What experience or training did the researcher have? | 1 Title page |
| *Relationship with participants* | | | |
| Relationship established | 6 | Was a relationship established prior to study commencement? | 6 Sampling procedure |
| Participant knowledge of the interviewer | 7 | What did the participants know about the researcher? e.g. personal goals, reasons for doing the research | Not specified |
| Interviewer characteristics | 8 | What characteristics were reported about the inter viewer/facilitator? e.g. Bias, assumptions, reasons and interests in the research topic | 8 Data analysis and rigour |
| **Domain 2: Study design** |  |  |  |
| *Theoretical framework* |  |  |  |
| Methodological orientation and Theory | 9 | What methodological orientation was stated to underpin the study? e.g.  grounded theory, discourse analysis, ethnography, phenomenology, content analysis | 6 Methodological framework |
| *Participant selection* |  |  |  |
| Sampling | 10 | How were participants selected? e.g. purposive, convenience, consecutive, snowball | 6-7 Sampling procedure |
| Method of approach | 11 | How were participants approached? e.g. face-to-face, telephone, mail, email | 6-7 Sampling procedure |
| Sample size | 12 | How many participants were in the study? | 9-10 Result and Table 1 |
| Non-participation | 13 | How many people refused to participate or dropped out? Reasons? | 9 Result |
| *Setting* |  |  |  |
| Setting of data collection | 14 | Where was the data collected? e.g. home, clinic, workplace | 7 Data collection |
| Presence of nonparticipants | 15 | Was anyone else present besides the participants and researchers? | Not specified |
| Description of sample | 16 | What are the important characteristics of the sample? e.g. demographic data, date | 9-10 Result and Table 1 |
| *Data collection* |  |  |  |
| Interview guide | 17 | Were questions, prompts, guides provided by the authors? Was it pilot tested? | 7-8 data collection |
| Repeat interviews | 18 | Were repeat inter views carried out? If yes, how many? | n.a. |
| Audio/visual recording | 19 | Did the research use audio or visual recording to collect the data? | 8 Data analysis and rigour |
| Field notes | 20 | Were field notes made during and/or after the interview or focus group? | 8 Data analysis and rigour |
| Duration | 21 | What was the duration of the interviews or focus group? | Not specified |
| Data saturation | 22 | Was data saturation discussed? | 8 Data analysis and rigour |
| Transcripts returned | 23 | Were transcripts returned to participants for comment and/or correction? | Not specified |
| **Topic** | **Item No.** | **Guide Questions/Description** | **Reported on Page No.** |
| **Domain 3: analysis and findings** | | | |
| *Data analysis* |  |  |  |
| Number of data coders | 24 | How many data coders coded the data? | 8 Data analysis and rigour |
| Description of the coding tree | 25 | Did authors provide a description of the coding tree? | 8-9 Explanatory theoretical model and  Table 2 |
| Derivation of themes | 26 | Were themes identified in advance or derived from the data? | 10 and 13 Explanatory theoretical model and  Table 2 |
| Software | 27 | What software, if applicable, was used to manage the data? | n.a. |
| Participant checking | 28 | Did participants provide feedback on the findings? | n.a. |
| *Reporting* |  |  |  |
| Quotations presented | 29 | Were participant quotations presented to illustrate the themes/findings?  Was each quotation identified? e.g. participant number | 10 Explanatory theoretical model and  Table 2 |
| Data and findings consistent | 30 | Was there consistency between the data presented and the findings? | 13-15 Discussion |
| Clarity of major themes | 31 | Were major themes clearly presented in the findings? | 13-15 Discussion |
| Clarity of minor themes | 32 | Is there a description of diverse cases or discussion of minor themes? | 13-15 Discussion |

Developed from: Tong A, Sainsbury P, Craig J. Consolidated criteria for reporting qualitative research (COREQ): a 32-item checklist for interviews and focus groups. *International Journal for Quality in Health Care*. 2007. Volume 19, Number 6: pp. 349 – 357

**Once you have completed this checklist, please save a copy and upload it as part of your submission. DO NOT** **include this checklist as part of the main manuscript document. It must be uploaded as a separate file.**
