# Supplementary material for: “Like before, but not exactly”: the Qualy-REACT qualitative inquiry into the lived experience of long COVID
Source: BMC Public Health. 2022 Mar 28;22:599. doi: 10.1186/s12889-022-13035-w (PMC8960224; doi:10.1186/s12889-022-13035-w)
Supplement: Supplementary file 2 — Additional file 2. Study Consent Form. [file 12889_2022_13035_MOESM2_ESM.pdf]

**Consent Form**

**REHABILITATION NEEDS AFTER HOSPITALISATION FOR CORONAVIRUS DISEASE 2019:**

**AN OBSERVATIONAL STUDY**

***(Rehabilitation needs after coronavirus-19 (REACT) hospital treatment: an observational study)***

I ____________________________________________________________

DECLARE

That I have received from ______________________________________ on ___/___/_______

comprehensive explanations regarding the request for participation in the study in question, as set out in the information sheet, a copy of which was delivered to me on ___/___/.

date ___/___/_______

To the best of my knowledge, I declare

- I have been informed about the aims, procedures, duration of this study sponsored by the by the Department of Physical Medicine and Rehabilitation
- I have been provided with a summary of the information relating to the characteristics of the study, that I was able to discuss these explanations, that I was able to ask any questions I felt necessary and that I received satisfactory answers
- To be aware that I am free to refuse to participate in the study and that I may withdraw my consent at any time during the duration of the study
- My participation in the study is completely voluntary
- I have been informed and I agree that my data may be made available to not only to the study managers and their delegates, but also to national and international health authorities and the Ethics Committee, if requested
- I am informed that my data may be communicated to national and international scientific congresses national and international scientific congresses or publication for scientific reasons in national and and international medical journals, but that in any case my identity will be protected by confidentiality (i.e. the data will always be always used in ANONYMOUS and AGGREGATED form)
- I □ Agree Do □ not agree that my GP is informed
- That I have been given a copy of this consent to hold

<u>By signing this form I agree to participate in the above mentioned study.</u>

| ___________________________ | _________________ | ___________________________ |
| --- | --- | --- |
| Name of Participant | Date | Signature |
| ___________________________ | _________________ | ___________________________ |
| Name of Resarcher | Date | Signature |
